# The inhibitory effects of butein on cell proliferation and TNF-α-induced CCL2 release in racially different triple negative breast cancer cells

**DOI:** 10.1371/journal.pone.0215269

**Published:** 2019-10-30

**Authors:** Patricia Mendonca, Ainsley Horton, David Bauer, Samia Messeha, Karam F. A. Soliman

**Affiliations:** College of Pharmacy and Pharmaceutical Sciences, Florida A&M University, Tallahassee, Florida, United States of America; Duke University School of Medicine, UNITED STATES

## Abstract

Drug resistance is the leading cause of breast cancer-related mortality in women, and triple negative breast cancer (TNBC) is the most aggressive subtype, affecting African American women more aggressively compared to Caucasians women. Of all cancer-related deaths, 15 to 20% are associated with inflammation, where proinflammatory cytokines have been implicated in the tumorigenesis process. The current study investigated the effects of the polyphenolic compound butein (2′,3,4,4′-tetrahydroxychalcone) on cell proliferation and survival, as well as its modulatory effect on the release of proinflammatory cytokines in MDA-MB-231 (Caucasian) and MDA-MB-468 (African American) TNBC cell. The results obtained showed that butein decreased cell viability in a time and dose-dependent manner, and after 72-h of treatment, the cell proliferation rate was reduced in both cell lines. In addition, butein was found to have higher potency in MDA-MB-468, exhibiting anti-proliferative effects in lower concentrations. Apoptosis assays demonstrated that butein (50 μM) increased apoptotic cells in MDA MB-468, showing 60% of the analyzed cells in the apoptotic phase, compared to 20% in MDA-MB-231 cells. Additionally, butein downregulated both protein and mRNA expression of the proinflammatory cytokine, CCL2, and IKBKE in TNFα-activated Caucasian cells, but not in African Americans. This study demonstrates butein potential in cancer cell suppression showing a higher cytotoxic, anti-proliferative, and apoptotic effects in African Americans, compared to Caucasians TNBC cells. It also reveals the butein inhibitory effect on CCL2 expression with a possible association with IKBKE downregulation in MDA-MB-231 cells only, indicating that Caucasians and African Americans TNBC cells respond differently to butein treatment. The obtained findings may provide an explanation regarding the poor therapeutic response in African American patients with advanced TNBC.

## Introduction

The increasing drug resistance in breast cancer therapy is the leading cause of cancer-related mortality in women [1]. In 2018, there was an estimated number of 266,000 new cases of invasive breast cancer to be diagnosed in the U.S., alongside 64,000 new cases of non-invasive breast cancer [2]. Breast cancer is classified into three major therapeutic subtypes: estrogen and/or progesterone receptor-positive (ER^+^, PR^+^), HER2^+^, and triple-negative breast cancer (TNBC) (lacking expression of ER, PR, and HER2) [3,4]. TNBC covers 15 to 20% of all breast cancers [5]. TNBC is more common in African American compared to other ethnic groups [6,7] and associated with a worse clinical outcome and higher mortality. [8,9]. TNBC subtypes respond differently to the treatment, challenging, even more, the development of target therapy with certain chemotherapeutics that may be safe and effective at the same time [4,10].

Compounds isolated from medicinal plants have been explored as a source of novel agents [11–13] with promising therapeutic potential with reduced adverse side effects. [14–16]. Butein (2’,3,4,4’-tetrahydroxychalcone) is a polyphenol compound found in several plants, including *Semecarpus anacardium*, *Dalbergia odorifera*, and *Rhus verniciflua* Stokes [17]. In Asian countries, butein has been used in herbal medicine formulations and as a food additive [18]. Also, butein exhibits a variety of pharmacological properties, including anti-inflammatory, antioxidative, and antimicrobial activities [19,20].

Breast cancer cell studies showed that butein inhibits ER^+^ MCF-7 cells growth [21], and blocks CXCL12-induced migration and invasion of human epidermal growth factor receptor 2 positive (HER2^+^) in SKBR-3 breast cancer cells by repressing NFқB-dependent CXCR4 expression [22]. Moreover, butein induced-apoptosis in MDA-MB-231, through ROS generation and ERK1/2 and p38MAPK dysregulation [23]. These findings show butein potential as a promising chemopreventive and chemotherapeutic agent for breast cancer [24].

In addition to breast cancer heterogeneity [25], tumor development and disease progression are influenced by the existence of the relationship between cancer and stromal cells at the tumor site [26–29], set by inflammatory cytokines, which are the crucial link between chronic inflammation and carcinogenesis [30–33]. Chronic incidence of TNF-α [34–36] and IL-1β [37–44] in tumors stimulate pro-tumoral effects in several cancers, showing that these two cytokines are potential targets for cancer therapy [39,45–47].

Despite the availability of evidence confirming butein effectiveness in tumor suppression, there is meager research information regarding its influence on the tumor cell response to proinflammatory cytokines, specifically TNF-α. In breast cancer, high concentrations of TNF-α can activate receptors and trigger a potent and persistent activation of NFқB signaling [48,49], epithelial-to-mesenchymal transition [50], and continuous release of diverse chemokines, including CCL2 and CCL5 [51]. These chemokines may initiate an inward migration of numerous leukocyte sub-populations (LPSs), including tumor-associated macrophages [52], myeloid-derived suppressor cells [53], tumor-associated neutrophils [54,55], T-regulatory [56], metastasis-associated macrophages, T helper IL-17-producing cells, and cancer-associated fibroblasts [57], which may bear CCR2 / CCR5 receptors, driving tumor aggression [36,58]. Therefore, chemokines are recognized as key trafficking molecules produced by cancer cells in response to TNF-α stimulation, and able of driving LSPs recruitment [31,59–61].

Although evidence in the literature show butein potential in protecting against and suppressing cancer, there are no studies to compare the effect of this compound on TNF-α-induced CCL2 release in Caucasian and African American breast cancer cell lines. Therefore, the present work was designed to investigate the effect of the polyphenol compound butein on TNF-α- activated ethnically different TNBC cells on cell viability, cell proliferation, and the release of proinflammatory cytokines.

## Materials and methods

### Cell lines, chemicals, and reagents

MDA-MB-231 (derived from Caucasian American TNBC) and MDA-MB-468 (derived from African American TNBC) were purchased from American Type Culture Collection (ATCC). Dulbecco’s modified Eagle’s medium (DMEM) high glucose; fetal bovine serum heat inactivated (FBS-HI), penicillin/streptomycin and Hank’s Balanced salt solution (HBSS) were obtained from Genesee Scientific (San Diego, CA, USA). Dimethyl sulfoxide (DMSO), butein, and Alamar Blue^®^ were purchased from Sigma-Aldrich Co. (St. Louis, MO, USA). Human cytokine antibody arrays (Cat# AAH-CYT-6-4), ELISA assays for MCP-1 (Cat# ELH-MCP1–1), Annexin V-FITC apoptosis Kit (Cat# 68FT-AnnV-S100), and tumor necrosis factor alpha (TNF-α) were purchased from RayBiotech (Norcross, Ga, USA). PCR primers, iScript advanced reverse transcriptase kit, and Bradford reagent were purchased from Bio-Rad (Hercules, CA, USA). DNA-free^™^ Kit (Cat # AM1907) from Life Technologies Inc. (Grand Island, NY, USA).). All reagents and plates for Western assays were purchased from ProteinSimple (San Jose, CA, USA). Primary antibodies were purchased from Cell Signaling (Danvers, MA, USA). The list of primary antibodies and their characteristics are described as follows (Table 1):

**Table 1:** List of primary antibodies used in western analysis.

Antibody	Type	Species Reactivity	Host/Isotype
IKK-epsilon	Polyclonal	human, mouse, rat	Rabbit/IgG
Phospho-IKK-epsilon	Monoclonal	Human	Rabbit/IgG

### Cell culture

MDA-MB-231 and MDA-MB-468 TNBC cells were cultured in DMEM supplemented with 10% FBS-HI and 1% penicillin (100 U/ml)/ streptomycin (0.1 mg/ml) and incubated in an atmosphere of 5% CO_2_ and 37°C. Cells were sub-cultured in T-75 flasks and grown to 90% confluency before setting the cells for each assay. Plating media for each experiment consisted of DMEM, with 2.5% of FBS-HI, with no penicillin/streptomycin.

### Cell viability and cell proliferation

Alamar Blue^®^ (Resazurin) assay was used to assess MDA-MB-231 and MDA-MB-468 cell viability and cell proliferation. Briefly, 96-well plates were seeded with cells at a density of 3×10^4^ cells/100 μl/well for cell viability and 5×10^3^ cell/well for cell proliferation studies and then incubated overnight in experimental media to attach. The next day, the cells were treated as follows: control (media only), control (cells + DMSO), and cells treated with different concentrations of butein (0.78–200 μM). Butein was dissolved in DMSO before dilution in the media, and the final concentration of DMSO did not exceed 0.1%. In the proliferative assay, Taxol (1 μM) was used as a positive control. The volume of 100 μl of each treatment was added to the plate-containing cells. Butein effect was measured after different periods of 24, 48, and 72 h incubation for cell viability, and after 72 h incubation for cell proliferation. The amount of 20 μl of Alamar Blue^®^ solution (0.5 mg/ml) was added to the plate and incubated again for 4 h. Quantitative analysis of dye conversion was measured at an excitation/emission of 550/580 nm wavelengths using a microplate reader Infinite M200 (Tecan Trading AG). Viable cells were able to reduce resazurin to resorufin, resulting in fluorescence changes. The fluorescent signal was proportional to the number of living cells in the sample, and the data were expressed as a percentage of alive untreated controls. Cell proliferation was calculated based on the percentage of cell growth observed in the control samples.

### Apoptosis assay

The effect of butein in inducing apoptosis was determined in MDA-MB-231 and MDA-MB-468 cells by using Annexin V-FITC Apoptosis assay Kit from RayBiotech. Briefly, each cell line was seeded at an initial concentration of 5×10^5^ cell/well in 6-well plates and incubated overnight. Cells were treated with butein at concentrations ranging between 0–200 μM in a final volume of 3 ml/well of experimental media to induce apoptosis. Control cells were exposed to DMSO at a concentration < 0.1%. After 24 h incubation period, controls and treated cells from each well were harvested, pelleted, and washed with PBS. According to the manufacture’s protocol, the cell pellets were resuspended in 500 μl of 1X Annexin -V binding buffer, then labeled with 5 μl of Annexin V-FITC, and 5 μl propidium iodide. The apoptotic effect was quantified within 5–10 min by FACSCalibur Flow cytometer (Becton Dickinson, San Jose, CA, USA). For each sample, 1 × 10^4^ cells were examined, and CELLQuest software was used for data analysis.

### Human cytokine antibody array membrane

RayBiotech human cytokine antibody arrays were used to study the effect of butein on 60 cytokine proteins released by TNF-α-activated TNBC cells. Each experiment was performed in triplicate and according to the manufacturer’s instructions. Shortly, antibody-coated array membranes were first incubated for 30 min with 1 ml of blocking buffer. Then, blocking buffer was decanted and replaced with 1 ml supernatant from cells exposed to the different treatments for a 24-h period. Treatments consisted of control (cells + DMSO) samples, cells treated with butein (5 μM), TNF-α (40 ng/ml), and the combination of butein (5 μM) + TNF-α (40 ng/ml). Membranes were incubated overnight at 4°C with mild shaking. The next day, the media were decanted; membranes were washed, and subsequently incubated with 1 ml biotin-conjugated antibodies for 2 h. Lastly, biotin-conjugated antibodies were removed, and membranes were washed again and incubated with HRP-conjugated streptavidin for 2 h. In this assay chemiluminescent reagent was used and the image of spots was captured using a Flour-S Max Multi-imager (Bio-Rad Laboratories, Hercules, CA, USA), and the spot density was determined with Quantity One Software (Bio-Rad Laboratories, Hercules, CA). Excel-based data analysis was performed, using Human Cytokine Array software C1000 (CODE: S02-AAH-CYT-1000) from RayBiotech.

### Human CCL2 (MCP-1) ELISA quantification

Supernatants were obtained from cells exposed to the different treatments for a 24-h period. Treatments consisted of control (cells + DMSO), butein-treated, TNF-α-stimulated, and co-treated (butein (5 μM) + TNF-α (40 ng/ml) TNBC cells were collected and centrifuged at 1000 rpm for 4 min at 4°C. Specific ELISA assays for CCL2 (MCP-1) was performed following the manufacturer’s instructions. Shortly, 100 μl of supernatants from each sample and standards were added to 96 well plates pre-coated with capture antibody and incubated for 2.5 h at room temperature under shaking. After washing, 100 μl of prepared biotinylated antibody mixture was added to each well and incubated for 1 h. The mixture was decanted, and 100 μl streptavidin solution was added to each well and incubated for 45 min. Substrate reagent (100 μl) was then pipetted into each well and incubated for 30 min, followed by the addition of 50 μl of stop solution. Samples were assayed at an optical density of 450 nm using Synergy HTX Multi-Reader (BioTek, USA).

### Real time polymerase chain reaction (RT-PCR)

#### RNA extraction

After cells were exposed to the different treatments for 24 h, cells were harvested, and the cell pellets were obtained. The treatments consisted of control (cells + DMSO), butein-treated (5 μM), TNF-α-stimulated (40 ng/ml) and co-treated with butein (5 μM) + TNF-α (40 ng/ml). First, the cell pellet was lysed with 1ml TRIzol reagent. Then, chloroform (0.2 ml) was added to the lysed samples; the tubes were shaken, incubated at 15–30°C for 2–3 min, and centrifuged at 10,000 rpm for 15 min at 2–8°C. Lysed samples (aqueous phase) were then transferred to a new tube, and mixed with 0.5 ml of isopropyl alcohol for RNA precipitation. After incubation (15 min), samples were centrifuged, the supernatant was removed, the RNA pellets were washed with 75% ethanol (by inverting the tubes carefully), and then centrifuged at 7,500 rpm for 5 min at 2–8°C. The RNA pellet was dried (room temperature), dissolved in RNase-free water, and incubated on ice (30 min). Finally, using Nanodrop (Thermo Fischer Scientific, Wilmington, DE, USA), RNA purity and quantity were determined.

#### cDNA synthesis and RT-PCR

The cDNA strands were synthesized from the mRNA using iScript advanced reverse transcriptase from Bio-Rad. A solution of 4 μl of the 5X iScript advanced reaction mix (containing primers), 1 μl of reverse transcriptase, 7.5 μl of the sample (1.5 μg/reaction), and 7.5 μl of water was combined in a 0.2 ml tubes, in a total volume of 20 μl. The thermal cycling program for the reverse transcription included two steps: 46°C for 20 min and then 95°C for 1 min. RT-PCR amplification was performed following the manufacturer protocol (Bio-Rad). A 1 μl of the sample (200 ng cDNA/reaction), 10μl of the master mix, 1 μl of primer, and 8 μl of water were combined into each well. The thermal cycling process included an initial hold step at 95°C for 2 min and denaturation at 95°C for 10 sec, followed by 39 cycles of 60°C for 30 sec (annealing/extension), and 65°C—95°C for 5 sec/step (melting curve) using the Bio-Rad CFX96 Real-Time System (Hercules, CA, USA). The selected primers were specific to each gene of interest. The UniqueAssay ID for *CCL2/MCP1* primer was qHsaCID0011608, and the for *IKBKE* primer was qHsaCID0014831.

### Capillary electrophoresis western analysis

Cells were exposed to different treatments for 24 h. The treatments consisted of control (cells + DMSO), butein-treated (5 μM), TNF-α-stimulated (40 ng/ml) and co-treated with butein (5 μM) + TNF-α (40 ng/ml). The next day, cells were harvested, washed twice with cold PBS, and centrifuged to obtain the cell pellet. Cells were then lysed with buffer containing protease inhibitor cocktail (total proteins) or protease plus phosphatase inhibitor cocktail (phosphorylated proteins). The concentration of protein was measured using Bradford reagent. Standards (5 μl) in concentrations ranging from 0 to 2 mg/ml or samples (5 μl) and 200 μl of protein assay reagent were added to the 96-well plate. Using a Synergy HTX Multi-Reader (BioTek, USA), the concentration of proteins was measured at 595 nm wavelength. Total and phosphorylated protein expression was determined using capillary electrophoresis western analysis (Wes, ProteinSimple, San Jose, CA, USA). Reagents and protocol for the assay were provided by ProteinSimple. First, the concentrations of antibody and protein to be used in the experiments were optimized by being tested in 3 different concentrations. From there, a specific concentration of antibody and protein was selected for further tests. Briefly, the protein samples (concentration: 0.13 mg/ml) were mixed with sample buffer, fluorescent molecular weight markers, dithiothreitol, and left in a heat block at 95°C for 5 min. The microplate was then loaded with blocking buffer, antibody (dilution 1:125), secondary antibody, chemiluminescent substrate, separation and stacking matrices, followed by centrifugation to remove bubbles. The microplate was placed in the instrument, and the electrophoresis and immunodetection occurred through the capillary system. This reaction identifies specific proteins by using primary and secondary antibodies and a chemiluminescent substrate. The chemiluminescence reaction and the digital image were analyzed by the software (ProteinSimple Compass).

### Statistical analysis

Data analysis was performed using GraphPad Prism (version 6.07) (San Diego, CA, USA). All data points are expressed as the mean ± S.E.M. from at least 2 independent experiments. For the viability studies, the IC_50_ was determined by nonlinear regression with R^2^ best fit and lowest 95% confidence interval. Statistically significant differences between different groups in the experiments was assessed using a one-way ANOVA, followed by Dunnett’s multiple comparison tests (*P < 0.05, **P < 0.01, ***P < 0.001, ****P < 0.0001, and ns = p > 0.05). Gene expression was analyzed using the CFX 3.1 Manager software (Bio-Rad, Hercules, CA). Protein expression using capillary electrophoresis western was analyzed by ProteinSimple Compass software (San Jose, CA, USA).

## Results

**Fig 1:**
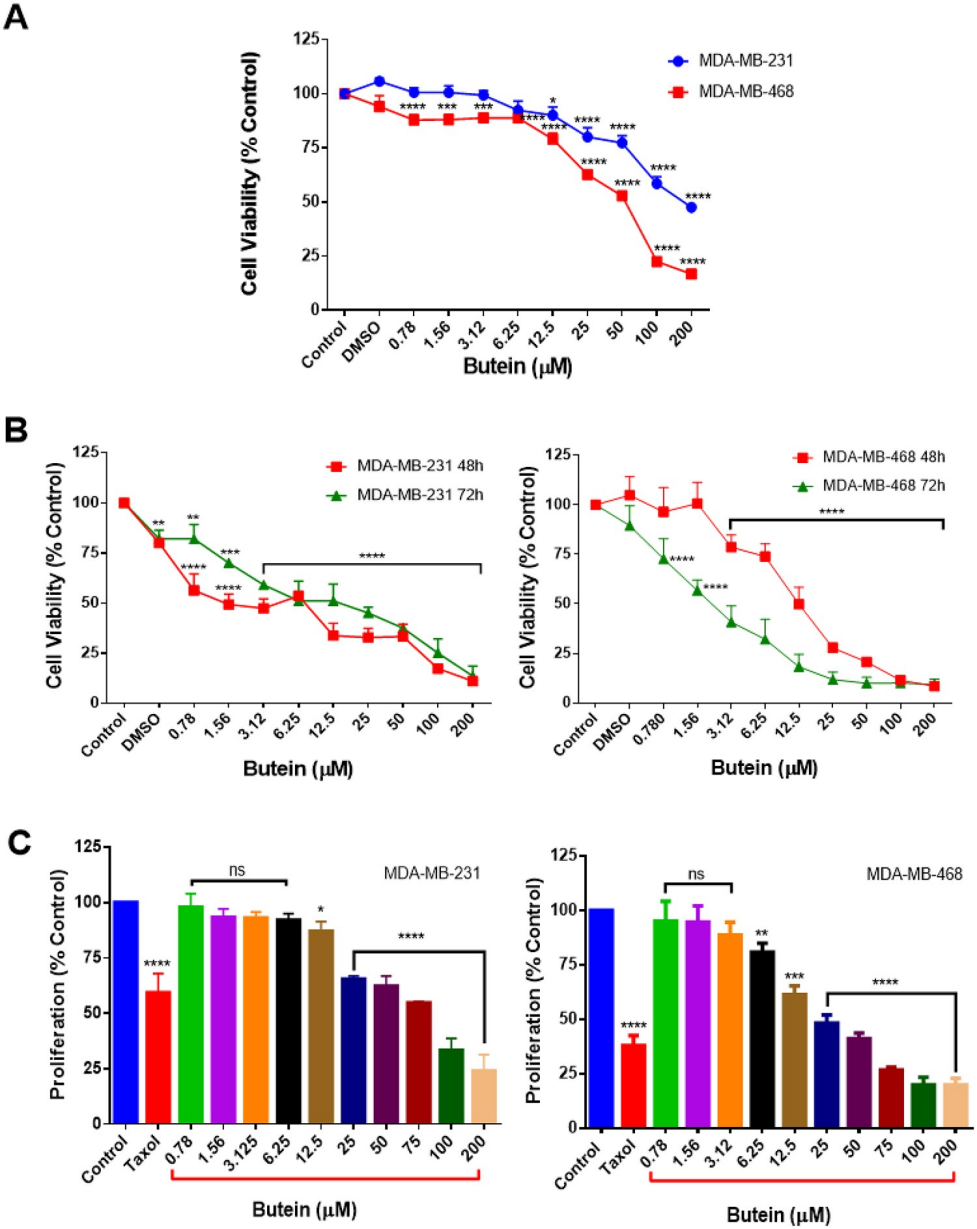
The effect of butein on cell viability and proliferation in MDA-MB-231 and MDA-MB-468 TNBC cells. Butein tested concentrations ranged from 0.78–200 μM. All experiments were performed at least 3 times with n = 5 and kept at 5% CO_2_ and 37°C. The cytotoxic effect was measured after 24 (**A**), 48 and 72-h (**B**), and the anti-proliferative effect after the 72-h treatment period (**C**). For both assays, cells were also treated with DMSO (<0.1%). For the proliferation assay, Taxol (1 μM) was used as a positive control. The data are presented as the mean ± S.E.M. Statistically significant differences between control vs. treatments were evaluated by a one-way ANOVA, followed by Dunnett’s multiple comparison tests. *p < 0.05, **p < 0.01, ***p < 0.001, ****p < 0.0001, ns = p > 0.05.

Butein effect on breast cancer cell viability was investigated in MDA-MB-231 and MDA-MB-468 cell lines after 24 h-treatment. Butein caused a concentration-dependent decrease in cell viability in both cell lines. Low concentrations of butein (from 0.78 to 6.25 μM) showed no cytotoxicity in MDA-MD-231; however, in MDA-MD-468, the decrease in cell viability was statistically significant (p < 0.0001) in the lowest concentration of 0.78 μM, compared to the control. The results indicate that butein effects on the two cell lines are different, causing higher cytotoxicity effect in MDA-MB-468 cells (Fig 1A). The cytotoxic effect of butein was also examined by incubating both cell lines with butein for 48 and 72 h. Results obtained indicate that cell viability rate was inversely correlated with the butein concentrations and exposure periods. At the 48-h incubation period, butein decreased the IC_50_s from 111.4 to 5.8 μM in MDA-MB-231, and from 33.8 to 8.7 μM in MDA-MB-468 cells. Further decrease in the cell viability was also measured at 72-h incubation period, reducing IC_50_s to 5.4 and 1.8 μM in MDA-MB-231 and MDA-MB-468, respectively (Fig 1B).

The anti-proliferation assays, based on the resazurin reduction, were performed to determine the potency of butein in inhibiting cell growth of both cell lines in comparison to the standard chemotherapy drug Taxol. Butein anti-proliferative effect was investigated through the measurement of the metabolic activity of the cells and their capacity to reduce resazurin after a 72-h period of incubation. The cells were treated with butein at concentrations ranging from 0.78 to 200 μM. In both cell lines, measurements of the proliferation rate after 72-h exposure time showed significant inhibition compared to the rate in the control groups. The decrease in cell proliferation rate was detected in a dose-dependent manner. In MDA-MB-468, butein started exerting its effect in a lower concentration (6.25 μM), compared to MDA-MB-231 cells (12.5 μM). However, in the highest concentration of 200 μM, butein inhibited over 70% of cell growth in both cell lines, presenting no significant difference in the anti-proliferative effect comparing MDA-MB-231 and MDA-MB-468 cells (p = 0.2785). Similar to Taxol after the 72-h treatment period, butein reduced breast cancer cells growth and showed its potency as an anti- proliferative agent (Fig 1C).

**Fig 2:**
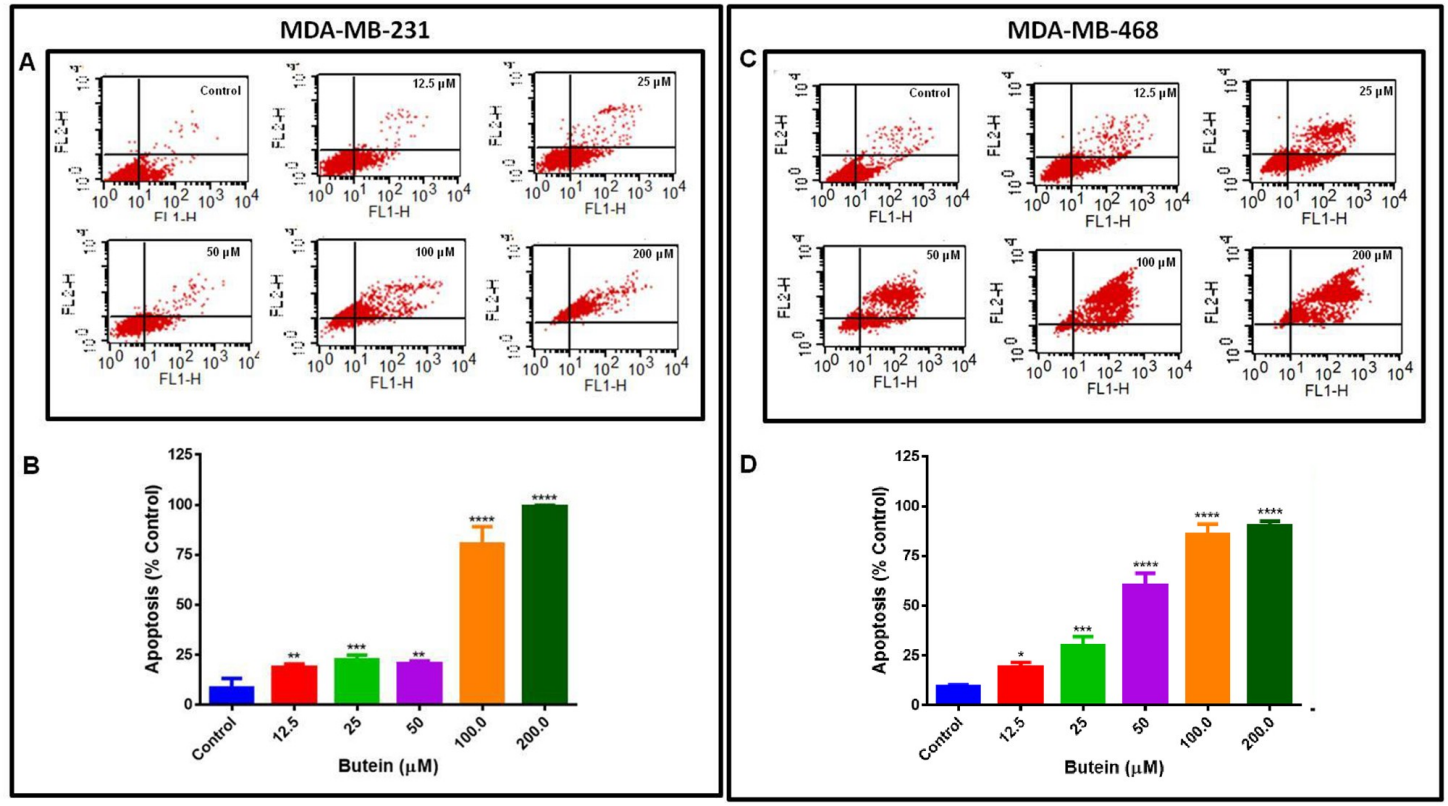
The apoptotic effect of butein in MDA-MB-231 and MDA-MB-468 TNBC cell lines. Cells were exposed to butein at concentrations ranging from 12.5–200 μM for 24 h, and control cells were treated with DMSO (< 0.1%). Apoptotic effect was determined by flow cytometry using Annexin V-FITC kit and FACSCalibur Flow cytometer to analyze the percentage of the apoptotic cells compared to the control cells. **A** and **C** represent the scatter plots for each one of the cell lines showing the movement of cells from the resting to the apoptotic state, and **B** and **D** show the percentage of apoptosis compared to the control group. The results represent the mean ± S.E.M. of two independent studies (n = 3). Statistically significant differences between control vs. treatments were evaluated by a one-way ANOVA, followed by Dunnett’s multiple comparison tests. *p < 0.05, **p < 0.01, ***p < 0.001, ****p < 0.0001.

The apoptotic effect of butein was determined by flow cytometry using Annexin V-FITC/PI staining in cells exposed to butein for 24 h. Annexin V has a high affinity for the phospholipid phosphatidylserine. The phospholipid translocation is followed by the loss of membrane integrity, accompanied by later stages of cell death resulting from either apoptotic or necrotic processes. The results showed that the percentage of viable cells decreased in the presence of butein in both cell lines (Fig 2A and 2C). In MDA-MB-231 cells, there was a significant increase of early apoptosis and a progressive increase of late apoptosis with increasing concentrations of butein. Treatment with concentrations of 12.5 (lowest) and 200 μM (highest) of butein increased the percentage of apoptotic cells (early and late) from 18.7 ± 1.8% to 99.4 ± 0.37% (Fig 2B). Moreover, MDA-MB-468 cells were also sensitive to butein, presenting apoptosis in 90.1 ± 1.44% of the cells at the highest concentration of butein (200 μM) after 24-h treatment (Fig 2D). Comparing butein effect in both cell lines, we observed that in the higher concentrations of 100, and 200 μM, butein effect was very similar in both cell lines, but in the concentration of 50 μM, butein effect was significantly higher in MDA-MB-468 cells (p<0.001) compared to MDA-MB-231 cells.

**Fig 3:**
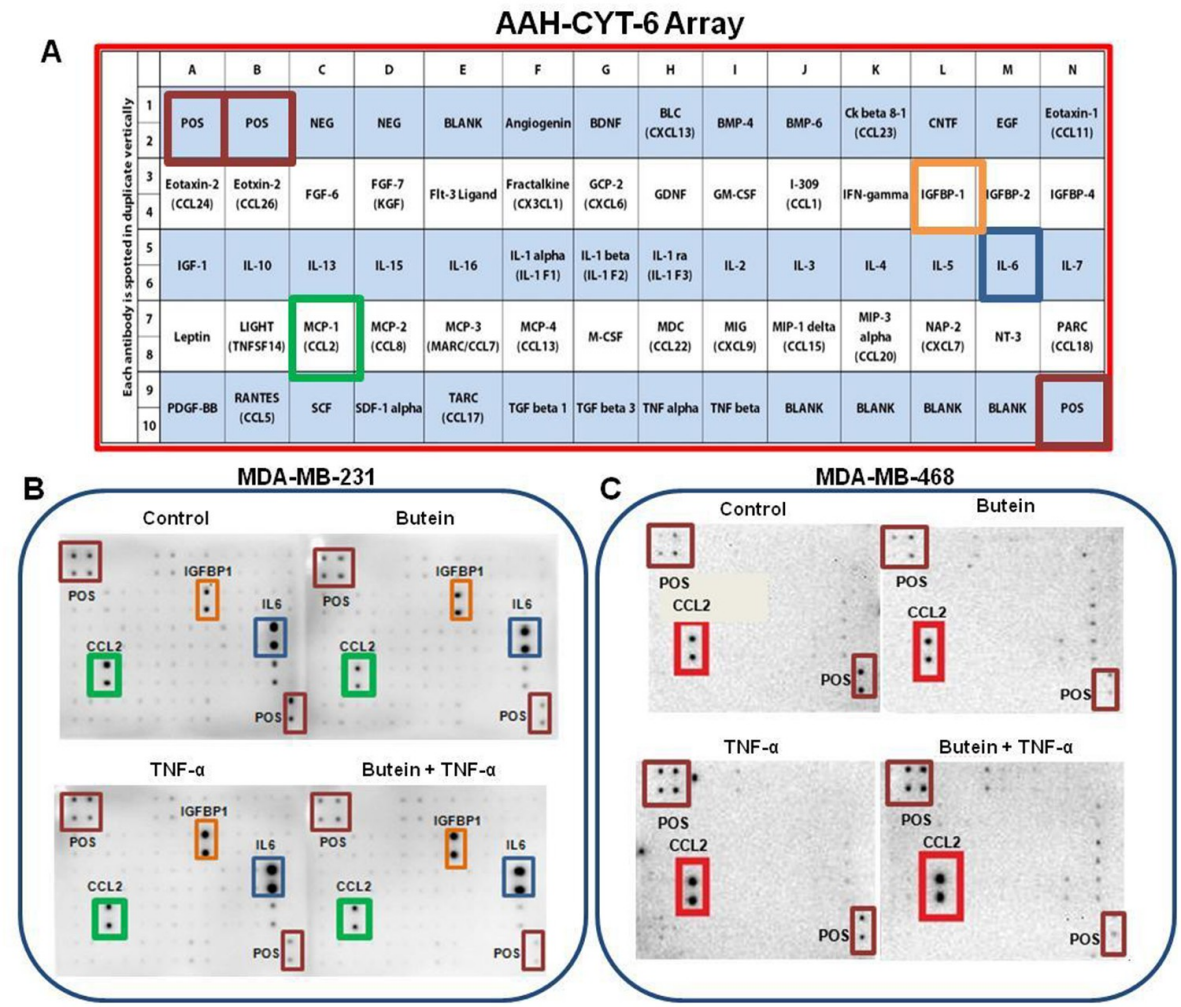
The effect of butein on cytokine expression in TNF-α-activated MDA-MB-231 and MDA-MB-468 TNBC cells (n = 3). **A-** Array layout used to assess chemokines/cytokines expression in the cell-free supernatants, showing the cytokines map, and highlighting CCL2 (MCP1), IGFBP-1, IL-6, and positive controls. **B and C**—Arrays with chemiluminescent spot intensity of supernatants derived from Caucasian breast cancer and African American cells showing cytokine changed expression after treatments. Blots represent the supernatants of 4 treatments: control (cells + DMSO), butein (5 μM), TNF-α (40 ng/ml), and butein (5 μM) + TNF-α (40 ng/ml) after 24-h treatment period.

**Fig 4:**
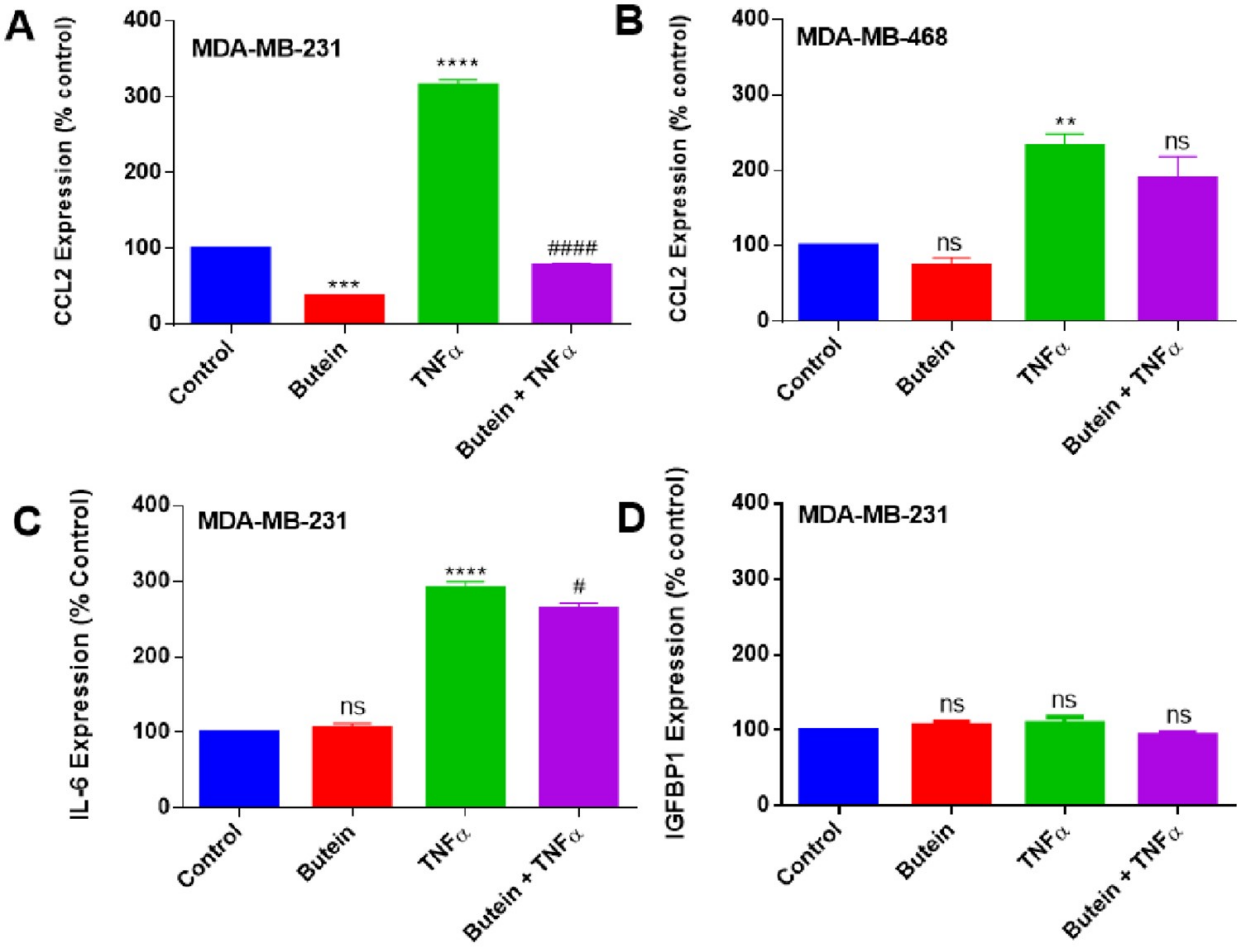
Normalized protein expression of CCL2, IL-6, and IGFBP1 (A, C, D) in MDA-MB-231 and (B) CCL2 in MDA-MB-468 TNBC cells. Data represent normalized dot spot intensities from the cytokine arrays calculated based on the positive controls found in the corners of each one of the membranes using RAYBIO^®^ANALYSIS software (RayBiotech). Data are expressed as % of control arrays (mean ± S.E.M. n = 3), representing 4 treatments: control (cells + DMSO), butein (5 μM), TNF-α (40 ng/ml), and butein (5 μM) + TNF-α (40 ng/ml). Statistically significant differences between control vs. butein and TNF-α (*)and TNF-α vs. butein + TNF-α (^#^) were evaluated by a one-way ANOVA, followed by Dunnett’s multiple comparison tests. **p < 0.01, ****p < 0.0001, ^#^p < 0.05, ns = p > 0.05.

In order to evaluate the relationship between the anti-cancer effects of butein treatment and its inhibitory effect on TNFα-activated proinflammatory cytokines, a semi-quantitative analysis using human antibody arrays was performed (Fig 3A). The results showed that TNF-α induced the upregulation of three specific cytokines: chemokine (C-C motif) ligand 2 (CCL2/MCP-1), insulin-like growth factor-binding protein 1 (IGFBP1), and interleukin-6 (IL-6) in MDA-MB-231 cells, although CCL2 was the only one upregulated in its counterpart MDA-MB-468 (Fig 3B and 3C). Butein presented a different effect in the two cell lines examined, inhibiting CCL2 expression in Caucasian, but not in African American cells. A dot blot intensity analysis of the arrays was performed using Quantity One software (Bio-Rad), and then each one of the dot spot intensities was normalized according to the positive controls found in the corners of each one of the membranes using RAYBIO ANALYSIS software (RayBiotech). The results obtained from TNF-α-stimulated cells and cells co-treated with butein and TNF-α show that butein attenuated TNF-α-induced CCL2 release significantly in MDA-MB-231 (4-fold inhibition), but not in MDA-MB-468 (Fig 4A and 4B). Normalized results also showed that butein treatment slightly inhibited the release of IL-6 in MDA-MB-231 cells, but there was no significant effect in the expression of IGFBP1 (Fig 4C and 4D).

**Fig 5:**
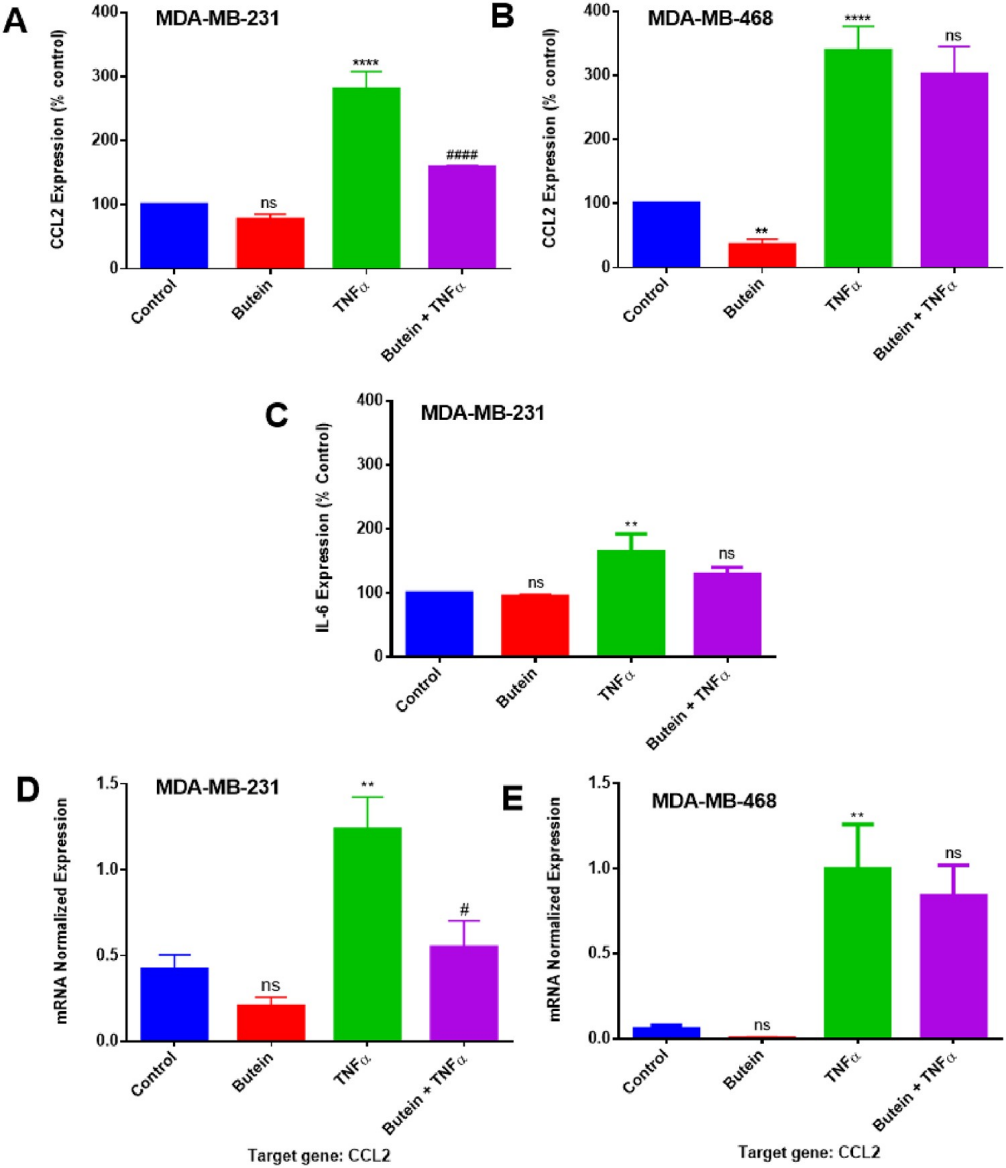
ELISA protein expression and mRNA quantification in MDA-MB-231 and MDA-MB-468 TNBC cells. The effect of butein (5 μM) on CCL2 (MCP1) and IL-6 protein expression in TNF-α stimulated MDA-MB-231 (**A,C**) and on CCL2 protein expression in TNF-α stimulated MDA-MB-468 cells (**B**). In **D** and **E**, the effect of butein in *CCL2* mRNA quantification in Caucasians and African American TNBC cells, respectively. Each data point represents the mean ± S.E.M. of three independent experiments (n = 3), representing 4 treatments: control (cells + DMSO), butein (5 μM), TNF-α (40 ng/ml), and butein (5 μM) + TNF-α (40 ng/ml). Statistically significant differences between control vs. butein and TNF-α (*)and TNF-α vs. butein + TNF-α (^#^) were evaluated by a one-way ANOVA, followed by Dunnett’s multiple comparison tests. **p < 0.01, ****p < 0.0001, ^####^p <0.001, ns = p > 0.05.

ELISA quantitative assays specific for CCL2 and IL-6 were used to validate the cytokine array findings. The results confirmed that TNF-α induces upregulation of CCL2 expression in both breast cancer cell lines and IL-6 expression in MDA-MB-231 cells. Butein treatment was able to downregulate CCL2 cytokine only in MDA-MB-231, with no significant effect on MDA-MB-468 cells, corroborating with the findings of butein effect using the cytokine arrays (Fig 5A and 5B). However, butein did not show any significant effect over the expression of IL-6 in MDA-MB-231 cells (Fig 5C).

Quantitative real-time PCR was used to investigate butein effect in *CCL2* gene expression in both breast cancer cell lines. The *CCL2* increased expression data had a similar trend as the results in the cytokine arrays and ELISA assays. TNF-α-induced *CCL2* expression was significant (p < 0.01) in both cell lines, compared to the control. Butein was effective in reducing *CCL2* expression significantly (p < 0.05) in MDA-MB-231 cells only, causing inhibition of more than 50% in mRNA expression (Fig 5D and 5E). These results indicate that the changes in *CCL2* expression caused by butein at the transcriptome level, follow the same pattern observed at the protein level.

**Fig 6:**
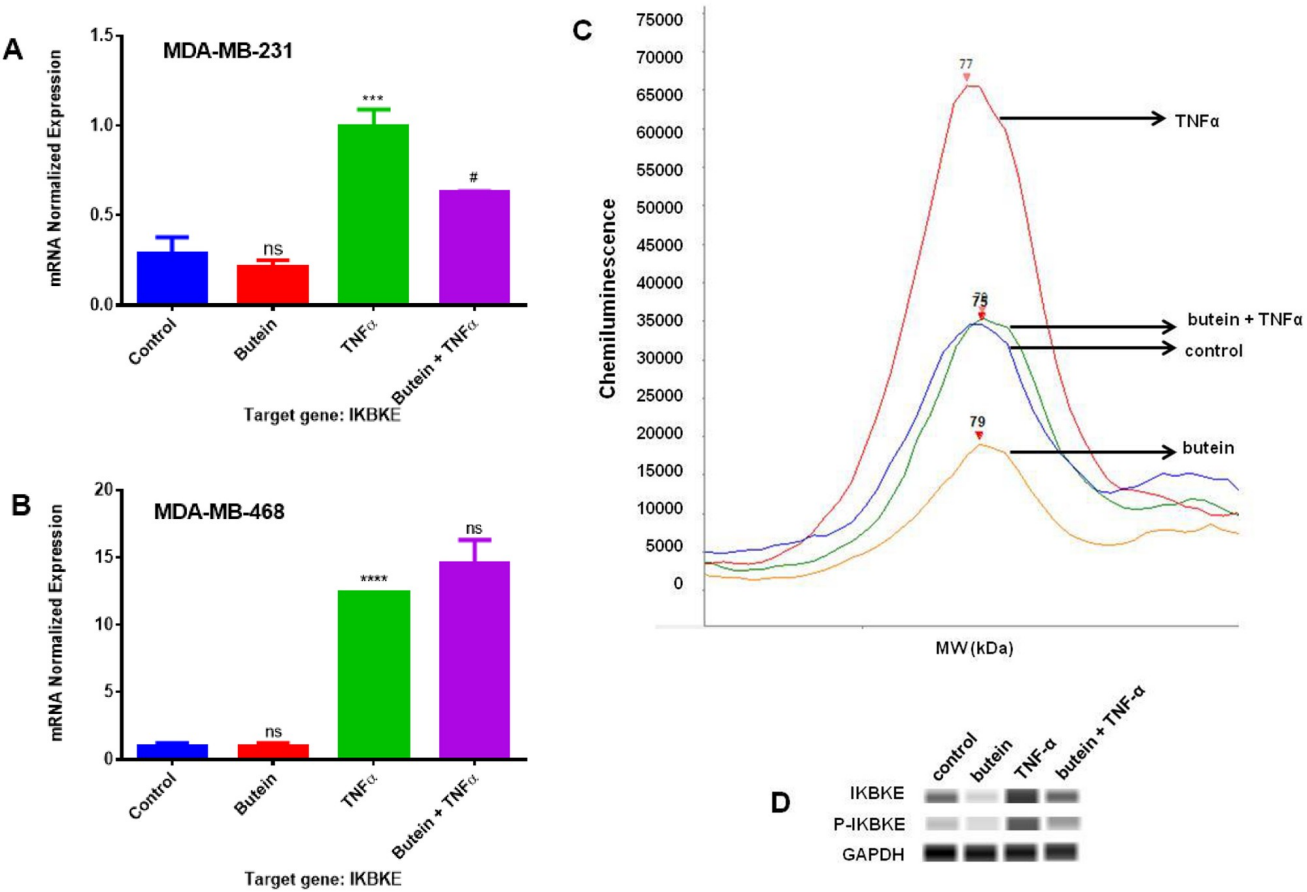
IKBKE mRNA and protein expression quantification in MDA-MB-231 and MDA-MB-468 TNBC cells. In **A** and **B**, the effect of butein (5 uM) in *IKBKE* normalized mRNA expression in TNF-α stimulated MDA-MB-231 and MDA-MB-468 cells. Each data point represents the mean ± S.E.M. of three independent studies (n = 3), representing 4 treatments: control (cells + DMSO), butein (5 μM), TNF-α (40 ng/ml), and butein (5 μM) + TNF-α (40 ng/ml). In **C**, electropherogram shows total IKBKE protein expression and the amount of chemiluminescence measured after Caucasian cells were exposed to the different treatments. In **D**, bands representing the protein expression after the 4 treatments (24 h) for total and phosphorylated IKBKE protein. Statistically significant differences between control vs. butein and TNF-α (*)and TNF-α vs. butein + TNF-α (^#^) were evaluated by a one-way ANOVA, followed by Dunnett’s multiple comparison tests. **p < 0.01, ***p < 0.001, ****p < 0.0001, ^#^p < 0.05, ns = p > 0.05.

To elucidate the possible signaling pathway related to the obtained findings, we investigated the changes in *IKBKE* mRNA expression. The results show that TNF-α upregulated *IKBKE* expression in both cells. TNF-α induced a 3.5 and 12.3-fold increase in mRNA expression in MDA-MB-231 and MDA-MB-468 cells, respectively, compared to the control. Although there was a higher expression of *IKBKE* in the TNF-α-stimulated MDA-MB-468 cells, butein co-treatment was only effective in MDA-MB-231 cells, inhibiting 37% of IKBKE mRNA expression (p < 0.05) (Fig 6A and 6B). To investigate the inhibitory effect of butein in IKBKE protein expression in MDA-MB-231 cells, we performed capillary electrophoresis western analysis with specific antibodies against total and phosphorylated IKBKE proteins. The results showed that TNF-α induced the expression of both of them in Caucasian cells, and their expression was significantly reduced when the activated MDA-MB-231 cells were treated with butein for 24 h (Fig 6C and 6D). These data demonstrate that IKBKE may be one of the NFқB signaling genes implicated in the TNF-α-induced *CCL2* release and its down-regulation by butein.

## Discussion

Polyphenolic compounds have received considerable attention for their use as a cancer chemopreventive and a chemotherapeutic agent. Previous *in vitro* studies showed butein cytotoxic and anti-proliferative effects on breast cancer cells, including MDA-MB-231 and MCF-7 [23,62], suggesting that butein might have similar effects in other breast cancer cell lines. However, there is no data comparing the effect of this compound in racially different TNBC cells. The current study shows butein anticancer properties in TNBC cells, specifically MDA-MB-231 and MDA-MB-468, representing Caucasians and African Americans. Overall the results obtained in our study provide more evidence for butein cytotoxicity towards both cell lines. However, the compound highly impacted MDA-MB-468 cells, in which lower concentrations were more effective in reducing cell viability (Fig 1A and 1B), and decreasing cell proliferation (Fig 1C). Also, the data show that butein induced apoptosis in both cell lines, increasing apoptotic cells ratio more effectively in MDA-MB-468 when lower concentrations were tested (Fig 2A, 2B, 2C and 2D).

Proliferative and anti-apoptotic effects have been described to be associated with NFқB signaling activation, which induces cell growth and arrests programmed cell death in multiple cell lines [63–65]. NFқB activation was found in ER negative breast cancer cell cultures [66], suggesting its role in proliferative pathways and cell death signals regulation [63–65]. NFқB can change cell homeostasis by inducing inflammatory processes, which have been described as a contributing factor in cancer development [67]. It is now clear that cell proliferation, by itself, doesn’t cause cancer. However, the uncontrolled proliferation in an environment rich in inflammatory cells, DNA damage inducers, and growth factors; all potentiate and/or increases the chances of tumor development [68].

Since there are many supporting evidence indicating the association of chronic inflammation with infection and irritation may promote the environment that leads to DNA lesions and tumor initiation [69], the current study investigated butein ability to inhibit TNF-α-mediated release of proinflammatory cytokines. The obtained findings in the current study show that butein attenuated the expression of CCL2, at both protein and mRNA levels in MDA-MB-231, but not in MDA-MB-468 cells (Figs 4A, 4B, 5A, 5B, 5D and 5E), demonstrating butein ability to inhibit CCL2 release only in Caucasians TNBC cells. CCL2 belongs to the C-C chemokines group and has been identified as an inflammatory modulator, which regulates macrophage recruitment during infection, the healing process, and autoimmune diseases. Through its CCR2 receptor affinity [70–72], it activates downstream signaling pathways, such as p42/44 MAPK, phospholipase C-γ, and protein kinase C. Elevated levels of CCL2 protein and mRNA expression are implicated in cancer, showing a high tumor grade and poor prognosis [73]. Moreover, CCL2 inhibition in mammary tumor-bearing mice decreased tumor growth, metastasis, macrophage recruitment, and angiogenesis, suggesting that this cytokine regulates tumor progression via a macrophage dependent mechanism [29,31,74–77]. Meanwhile, Fang *et al*. (2012) demonstrated that CCL2 treatment decreased apoptosis caused by serum deprivation, gentamicin or 5-FU treatment in mouse and human mammary carcinoma cells (MDA-MB-231), suggesting that CCL2 may induce pro-survival effects in human breast cancer cells [78]. Also, they show that CCL2 effect on cell survival is linked to an increase of phosphorylation of Smad3 and p42/44 MAPK proteins [78].

The findings of our work demonstrated butein ability to induce apoptosis and inhibit TNF-α-induced CCL2 release. Further research is still needed to confirm the association between cell survival regulation and CCL2 inhibition in MDA-MB-231 cells. Our data corroborate with previous literature studies showing the significant role of CCL2 signaling in breast cancer cells [79,80] and indicates that targeting CCL2 signaling pathway may affect various mechanisms involved in cancer progression, hence representing an attractive therapeutic target [78]. The present study also determined that butein inhibitory effect on CCL2 expression was only effective in MDA-MB-231 cells, suggesting that the apoptotic effect in MDA-MB-468 cells is not associated with CCL2 regulation.

Our investigation showed that butein inhibitory effect on CCL2 expression in Caucasian cells might be attributed to its ability to downregulate IKBKE mRNA and protein expression (Fig 6A, 6B, 6C and 6D). IKBKE is a gene overexpressed in approximately 30% of human breast tumors [81] and represents an emerging link between cancer and inflammation [82]. It promotes cytokine release and pro-survival signaling through the activation of NFқB and JAK–STAT signaling pathways [83]. Using JAK inhibitors that also target IKBKE, Barbie *et al*. [83], verified that there was a decrease in the viability of TNBC cells with *IKBKE* increased levels. This gene also regulates survival signaling associated with NFқB pathway activation, enabling cell transformation [82,84]. Also, Bauer *et al*. [85] studied the association between the *IKBKE* gene and CCL2 release, showing that *IKBKE* downregulation attenuates CCL2 expression in MDA-MB-231 TNBC cells. Likewise, the data from our previous study [86] demonstrated that the natural compound plumbagin inhibited *IKBKE* gene expression and consequent release of CCL2 in TNF-α-induced MDA-MB-231 cells, strengthening the potential association between *IKBKE* and CCL2 expression.

**Fig 7:**
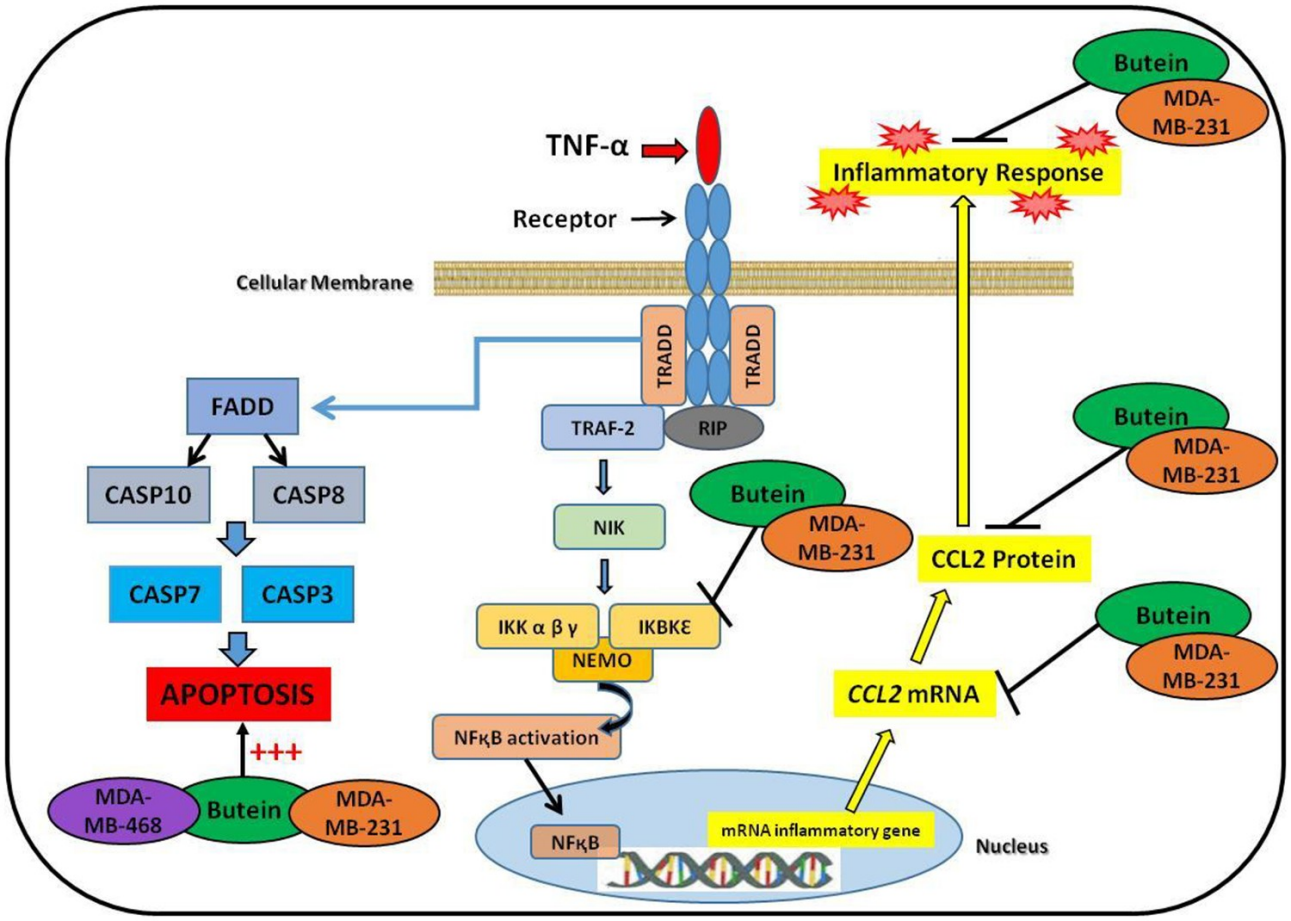
Proposed mechanism of butein effect in MDA-MB-231 and MDA-MB-468 TNBC cells. The diagram shows butein inhibitory effect in TNF-α-stimulated CCL2 expression at mRNA and protein level, attenuating *IKBKE* expression as a possible molecular mechanism in Caucasian cells; in addition to a possible signaling pathway for butein apoptotic effects in MDA-MB-231 and MDA-MB-468 TNBC cells.

In summary, the present investigation demonstrates butein potential in cancer suppression of the two different TNBC cell lines: MDA-MB-231 and MDA-MB-468. Butein showed higher cytotoxicity, anti-proliferative, and apoptotic effects in MDA-MB-468, compared to MDA-MB-231. Additionally, butein downregulated both protein and mRNA expression of TNF-α-stimulated CCL2 release in Caucasian cells but not in African Americans. Moreover, the results elucidated one, out of many molecular mechanisms that may be involved in CCL2 downregulation, showing butein inhibitory effect on *IKBKE* mRNA and protein expression in MDA-MB-231 cells (Fig 7). Therefore, the obtained findings indicate that butein might be a potential candidate for breast cancer therapy targeting CCL2 in Caucasians and may also provide an explanation regarding the poor response to therapy in African American patients with advance TNBC.
